# From fairytale to clinical practice: A response to commentaries

**DOI:** 10.1111/add.70206

**Published:** 2025-10-16

**Authors:** Mariana Gonzalez Utrilla, Edward Chesney, Joanne Neale, Nicola Metrebian, Nicola Kalk, Arne Kristian Skulberg, Paul Dietze, Martin Smith, John Strang

**Affiliations:** ^1^ National Addiction Centre Institute of Psychiatry, Psychology and Neuroscience, King's College London London UK; ^2^ South London and Maudsley NHS Foundation Trust London UK; ^3^ Centre for Social Research in Health University of New South Wales Kensington Australia; ^4^ Division of Prehospital Services Oslo University Hospital Oslo Norway; ^5^ Department of Circulation and Medical Imaging Norwegian University of Science and Technology Trondheim Norway; ^6^ Disease Elimination Program Burnet Institute Melbourne Australia; ^7^ National Drug Research Institute and Enable Institute Curtin University Melbourne Australia; ^8^ Derbyshire Healthcare NHS Foundation Trust Derby UK

**Keywords:** community‐based interventions, dose titration, emergency response, fentanyl, harm reduction, naloxone, opioid overdose, overdose reversal, synthetic opioids, take‐home naloxone

*Evidence gaps in synthetic opioid overdose management require urgent research through observational studies, experimental investigations and clinical trials. Three priorities emerge: comprehensive naloxone training that emphasizes basic first aid, improved products ensuring accurate dose titration and simple teachable principles for lay responders to implement graded naloxone administration safely*.

We thank our colleagues for their responses to our article [1] in which we proposed a more nuanced approach to the emergency management of opioid overdose. We are pleased that it has generated thoughtful discussion and debate.

Wong *et al*. [2] raise the valid concern that evidence on responding to potent synthetic opioids is lacking, concluding that ‘the risk of inadequate reversal […] often outweighs the potential harms of over‐antagonism.’ We agree that there is a pressing need for improved evidence on responses to synthetic opioid overdose to inform real‐world, evidence‐based practice. There is an urgent need for clinical trials, observational studies and emergency medical services data analysis involving these drugs.

Coffin [3] supports our core argument for integrating tailored dosing strategies into overdose management protocols. We agree that over‐reliance on naloxone risks delaying other critical interventions, such as respiratory and cardiovascular support. Coffin also highlights the importance of timing, noting that fentanyl‐related mortality may reflect not only its potency, but also the rapid onset of its effects compared with heroin.

Similarly, Morgan and Walley [4] emphasize the importance of timely intervention and how dose selection is secondary to a more fundamental issue—whether a bystander is present and able to respond, particularly given that witnesses may not be present. On this basis, not consuming drugs alone should be a core in harm reduction strategies. Our research group is one of a number around the world developing real‐time overdose detection interventions to enable the earliest possible response [5].

We believe that there are three practical aspects that deserve emphasis. First, naloxone training in the community must include recognizing symptoms, basic first aid including airway and rescue breathing and alerting medical services before naloxone administration. Second, we need improved naloxone products that allow dose titration in community settings. Products designed to ensure individual incremental dosing could help bridge the gap between clinical and community environments. Recent pharmacokinetic studies have demonstrated significant inter‐individual variability in naloxone absorption, supporting the need for flexible dosing approaches [6, 7]. The development of naloxone formulations that permit stepwise administration could address both efficacy and safety concerns in community settings. Third, we should develop simple, teachable principles of dose titration for lay responders. Rather than complex protocols, basic training could focus on the initial assessment and then starting conservatively if the patient is not in extremis/still breathing, monitoring respiratory response, and understanding when additional doses are warranted. Evidence from emergency medical services suggests that trained responders can successfully implement graded naloxone administration, and similar principles could be adapted for community use [8, 9].

The evidence gaps identified by all commentators underscore the need for research that addresses real‐world application challenges. The design and conduct of randomized controlled trials during active overdose events is ethically challenging although several research groups have successfully conducted such trials [10, 11, 12, 13, 14, 15], and valuable conclusions can also be reached from observational studies, emergency medical services data analysis and carefully designed simulation studies to build the evidence base for optimal dosing strategies. Human laboratory studies of controlled opioid intoxication and reversal will also be important [16]. Recent analyses of naloxone distribution programmes have provided valuable insights into usage patterns and outcomes that can inform future product development [17].

The diversity of perspectives in these commentaries reflects the genuine complexity of optimizing overdose response in the synthetic opioid era. We believe this complexity justifies continued exploration of a range evidence‐based approaches that can be practically implemented across the range of settings where overdose response occurs.

The shared goal remains clear: saving lives through effective, compassionate intervention that minimizes both fatal outcomes and iatrogenic harm.

## DECLARATION OF INTERESTS

None.
